# The Pollution of Water Supplies

**Published:** 1897-11-27

**Authors:** 


### THE POLLUTION OF WATER SUPPLIES.

The great interest which has recently been excited in regard to the ever present possibility of pollution, which seems to hang over our public water supplies, has found expression in a paper read by Professor Corfield at the Sanitary Institute, and in a discussion at the Royal Medical and Chirurgical Society on "The Prevention of Enteric Fever," which was opened on Tuesday evening by Dr. Vivian Poore.

At the present stage of this discussion it is too early to enter on the real question at issue, viz., the prevention of the disease, but we may usefully refer to some of the points raised by Dr. Poore in his brilliant onslaught upon the existing state of affairs, and upon what, we suppose, he would call the present "fashion" in sanitary science.

The first thing is to recognise how many are the chances of pollution to which ordinary town water supplies are exposed.

Great faith has been placed upon the purity of water pumped from deep down in the chalk. The reputation of many of these wells, however, would seem to have depended largely on various more or less accidental circumstances, for, when brought to the test, the security which is imagined to attach to them, in consequence of the depth to which they are driven, is found in some instances to be illusory. Instances are given of the fouling of such wells by accidental leakage from sewers, by trickling from the surface of an adjacent sewage farm, and by leakages from foul ponds. The mischief of chalk is that it contains cracks and fissures, some of which extend for a long distance, so that it becomes very difficult to make sure that a well, however deep it may be, is not affected by some far distant source of contamination.

Some of the most remarkable instances of the infection of public water supplies are to be found in those cases in which the contamination has taken place from leakage into the pipe by which the water is distributed. Dr. Poore described with great vividness the proceedings which he said were going on in a well known and progressive suburb of London. The country roads were being drained and paved, water pipes were being laid down, and buildings were being erected on every side. One saw the water pipes being laid side by side with the drains only a short distance from each other, and from the surface of the road; and then one heard the rumble of the steam roller, grinding everything beneath it as if intent upon producing a commingling of their contents.

And no doubt what does happen in unnumbered instances is that the pipe is cracked and the drain is broken, and the water is befouled, and nobody knows anything about it until the befoulment happens to be tainted with the typhoid poison, when an epidemic incontinently appears.

There can be no doubt of the danger that attends a badly managed public water supply. The question is as to the remedy. Few would entirely disagree with Dr. Poore as to the advantage of disposing of domestic *débris* in the domestic garden, if that were possible. It is no doubt a fine ideal to be rid of drains and water supplies, and, if possible, of water rates; to draw from one's own well, and to dig all the rubbish into the earth within sight of the house from which it comes. If only we did but have a garden! Here comes in the criticism which Sir Richard Thorne applied to the whole argument. Admitting that the epidemicity of typhoid chiefly depends on public water supplies, this is a matter which has principally to do with towns. But the remedy proposed, viz., the disposal of excreta in the garden, is a matter concerning the country; the evil and the remedy concern different conditions of life, and the one does not touch the other.

Everyone recognises that sanitary appliances are not perfect. But they are getting better; the standard demanded is continually getting higher, and it would be unfair to condemn what is now being done to promote good drainage and pure water supplies because much of the early drainage was done badly, and many of the old water pipes are rotten.

Even with what has been done the deaths from typhoid fever are vastly less than they used to be. In years gone by the typhoid fever from which the country suffered was principally endemic, and it has been in the lessening of this endemic typhoid that the gain of the last thirty years has been principally seen. On the other hand, no doubt various great epidemics have occurred. These, however, have been the result not of water supply and drainage, but of defective systems of water supply and defective systems of drainage. The remedy, then, is not to revert to archaic methods, but to constantly improve our systems and remedy their defects.

